# Intraneural metastasis of gastric carcinoma leads to sciatic nerve palsy

**DOI:** 10.1186/1471-2407-12-313

**Published:** 2012-07-25

**Authors:** Jiro Ichikawa, Seiichi Matsumoto, Takashi Shimoji, Taisuke Tanizawa, Tabu Gokita, Keiko Hayakawa, Kaoru Aoki, Saori Ina, Hiroaki Kanda

**Affiliations:** 1 Department of Orthopaedic Surgery, Cancer Institute Hospital for Japanese Foundation for Cancer Research, 3-8-31 Ariake, Koto-ku, Tokyo, 135-8550, Japan; 2 Department of Pathology, Cancer Institute Hospital of Japanese Foundation for Cancer Research, 3-8-31 Ariake, Koto-ku, Tokyo, 135-8550, Japan

**Keywords:** Intraneural metastasis, Nerve palsy, Palliative surgery

## Abstract

**Background:**

Soft tissue metastases, in particular intraneural metastasis, from any carcinomas seldom occur. To our knowledge, no case of sciatic nerve palsy due to intraneural metastasis of gastric carcinoma is reported in the literature.

**Case presentation:**

A case is reported of a 82-year old woman with sciatic nerve palsy with intraneural metastasis of gastric carcinoma. Although she had undergone partial gastrectomy with T2b, N0, M0 two years ago and primary site was cured, she developed sciatic nerve palsy from the carcinoma metastasis directly to the nerve. Operative resection and Histological examination revealed poorly differentiated adenocarcinoma, the same as her primary site adenocarcinoma.

**Conclusions:**

Sciatica is usually caused by a herniated disc or spinal canal stenosis. Sciatic nerve palsy may be caused by nondiscogenic etiologies that may be either intrapelvic or extrapelvic. It is important to image the entire course of the nerve to distinguish these etiologies quickly. The longer the nerve compression the less likely a palsy will recover. Surgery is a good intervention that simultaneously obtains a tissue diagnosis and decompresses the nerve.

## Background

The common metastatic sites of gastric carcinoma are liver, lung, lymph nodes, and peritoneum^,^ whereas metastasis to soft tissue, in particular ‘nerve’, is extremely rare. Sciatica and Sciatic nerve palsy are usually caused by lumbar etiologies such as a herniated disc or spinal canal stenosis [1]. To our knowledge, Sciatic nerve palsy caused by intraneural metastasis of gastric carcinoma has never been described. Here we report a patient who had sciatic nerve palsy by intraneural metastasis of gastric carcinoma and was successfully treated by wide resection.

## Case presentation

**Figure 1:**
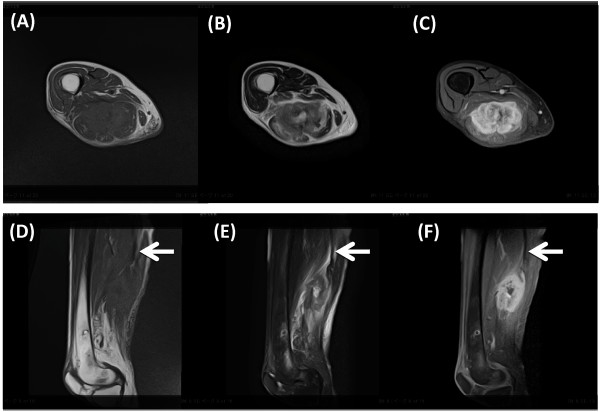
**MRI of the thigh.** Upper sections represent sagittal views, lower sections axial views. (**A**) (**D**) T1-weighted, (**B**) (**E**) T2-weighted, and (**C**) (**F**) enhanced T1-weighted images. MRI showed the mass arising from sciatic nerve (arrow) with low signal intensity on T1-weighted images, and heterogeneous high signal intensity on T2-weighted images and homogeneous high signal intensity on enhanced T1-weighted images.

**Figure 2:**
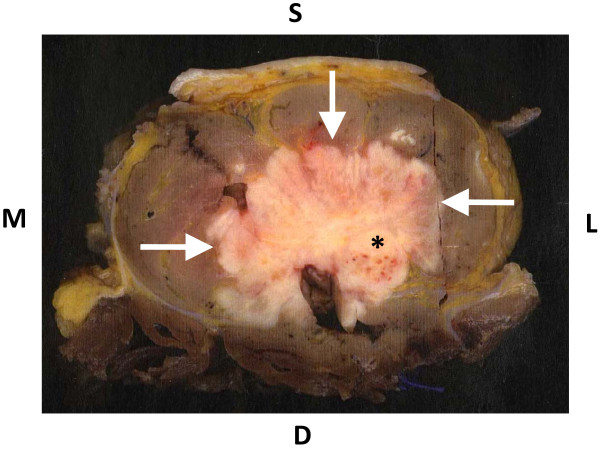
**Macroscopic finding of axial section.** The tumor (arrow) surrounded sciatic nerve (*) and compressed surrounding muscles. S;Superficial, D; Deep, M;Medial, L;Lateral.

**Figure 3:**
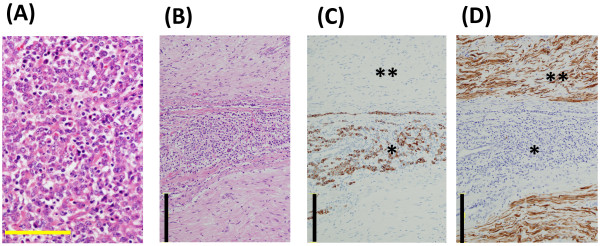
**Histology of the stomach (A;Primary site, H&E) and thigh (B; metastatic lesion H&E).** There are poorly differentiated adenocarcinoma in both lesion. Note nerve invasion of adenocarcinoma (**B**). Immunohistochemical study (**C**; Cytokeratine and **D**;S-100) clearly demonstrated nerve (* S-100 positive) invasion of poorly undifferentiated adenocarcinoma (** Cytokeratine positive).

A 82-year-old woman was admitted to our hospital because of right sciatic nerve palsy and a mass in the right posterior thigh. Symptoms of sciatica had begun 6 months ago and then she visited another hospital and X-ray and MRI of the lumbar spine was performed. These showed slight canal stenosis of the lumbar spine. Although she was treated conservatively with medications, the symptoms worsened and sciatic nerve palsy resulted 3 weeks before visit to our hospital. Physical examination revealed a soft tissue mass of posterior thigh with pain and a drop foot with concomitant sensory loss. The patella tendon reflex was normal but Achilles tendon reflex was negative. The sensory exam and reflexes and manual muscle testing in the left leg were normal. All routine blood tests were normal. She had undergone partial gastrectomy 2 years ago. Resection was complete and TNM stage was T2b, N0, M0. The size of tumor was 8.5 x 8 cm and histological examination revealed the tumor was a poorly differentiated adenocarcinoma with no regional lymph node metastasis. She didn’t receive adjuvant chemotherapy because of her age and her choice. Current Plain radiographs showed a radiolucent area in the posterior thigh without calcification and no changes in the femur. MRI (Figure 1) showed the mass with low signal intensity on T1-weighted images and heterogeneous high intensity on T2-weighted images and homogeneous high intensity on T1- weighted images with gadolinium enhancement was originated from sciatic nerve. We planed the surgical resection because her general condition was good and we confirmed local disease only, no evidence of another site of metastasis by CT. Surgical resection of the sciatic nerve mass was based on the assumption that the diagnosis was soft tissue sarcoma. Segmental resection of the sciatic nerve was performed because of complete paralysis and adequate margin. Intraoperative Macroscopic findings (Figure 2) revealed the sciatic nerve was tightly surrounded by the mass and the mass strongly compressed the peripheral muscles. Histological examination (Figure 3) showed poorly differentiated adenocarcinoma which was the same as her primary site adenocarcinoma and invaded the sciatic nerve. Based on these findings, we diagnosed sciatic nerve palsy due to intraneural metastasis of gastric carcinoma. She can now walk with a short leg brace and cane. She is free of recurrence in the posterior thigh without chemotherapy and radiation and remains metastasis free after 1 year follow-up.

## Discussion

Hematongenous soft tissue metastases seldom occur but are seen from lung carcinoma, renal carcinoma [2,3]. Intraneural metastasis from carcinoma is extremely rare. A few cases were reported including mammary carcinoma [4], lymphoma [4], renal carcinoma [5], and melanoma [6]. To the best of our knowledge, this report is the first description of a case of sciatic nerve palsy due to intraneural metastasis of gastric carcinoma.

Soft tissue metastasis including the muscles, tendons, ligaments, subcutaneous tissues, skin and nerve is very rare compared to lungs, liver, bones and lymph nodes. Several factors have been implicated in the rarity of soft tissue metastasis such as changes in pH, accumulation of metabolites, and local temperature at soft tissue sites [7] The organs with high frequency of metastasis are rich in capillary vessel and have a constant flow, whereas in soft tissue blood flow is variable and is influenced by adrenergic receptors and is subject to varying tissue pressure that may affect tumor implantation [7-9]. Another reason of rarity of intraneural metastasis by carcinoma is existence of ‘blood-nerve barrier’ which, similar to the blood–brain barrier, may prevent implantation of tumor cells by vascular channels [10].

Sciatica is a common condition, affecting as many as 40% of adults at their lives [11] and continual sciatica can finally result in sciatic nerve palsy. Although sciatica is usually caused by lumber disc hernia and lumber canal stenosis, it is not often but we should consider the nondiscogenic sciatica, which can be categorized as either intrapelvic and extrapelvic [11]. The causes of extrapelvic include aneurysms or pseudoaneurysms of gluteal artery [12-14], tumors [15], gluteal abscess [16], avulsion fracture of the ischial tuberosity [17], and paralabral cysts [11]. There are three ways by which tumor can influence the functional and structural integrity of nerve tissue; (1) the tumor can stretch the nerve trunk by pushing it without invading the sheath; (2) the tumor can compress or strangulate the nerve by engulfing it without genuine invasion of the sheath; (3) the tumor can perforate the nerve [10,18]. Our case is consistent with (3) because MRI, Macroscopic findings, Histological examination and clinical behavior totally supported the fact that metastatic gastric carcinoma directly invaded sciatic nerve and spread the surrounding muscles. Based on these findings, we concluded the cause of sciatic nerve palsy is not the invasion of soft tissue metastasis but direct intraneural metastasis.

Treatments including radiotherapy, chemotherapy and surgical excision are controversial [2] because prognosis for patients with soft tissue metastasis is poor and mean survival was only 8.4 months [3]. This is why the management of the soft tissue metastasis including intraneural metastasis depends on the clinical setting and the condition of the patients. In our case, although the patient was elderly, our decision for treatment was base on the below facts, 1) primary site was completely cured 2) no evidence of other site metastasis by CT 3) good general condition 4) possibility the mass could get larger and more painful in short time. Especially in the case of intraneural metastasis, surgical excision seems to be the only option [4-6] but we should be careful because of two opposite reasons 1) reductive excision means the higher rate of recurrence 2) curative excision means the larger damage and loss. Usually it is very difficult to decide which is harmless for patients, curative or reductive. In our case, we could easily emphasize the less recurrence induced by curative excision because sciatic nerve had already paralyzed. Our patient has benefitted from excision to provide the free survival but we should absolutely consider the two previous facts, 1) the success to prolong survival has been reported anecdotally after excision of solitary soft tissues masses from only renal [19] and, rarely, lung primaries [7]. 2) In addition, excision of lung and colon soft tissue metastases led to rapid local recurrence, regional lymph node spread and resulted in widespread dissemination of disease and death in short order [20-22].

## Conclusions

Here we report a rare case of sciatic nerve palsy due to intraneural metastasis of gastric carcinoma with successful treatment. We carefully differentiate the causes of sciatica and sciatic nerve palsy, which are divided between discogenic and nondiscogenic including the intrapelvic and extrapelvic. Although our strategy is that we choose the excision in the case of solitary metastasis, complete cure of primary site, good condition of patients, further clinical studies are needed to investigate our strategy and to establish the standard therapy for any types of soft tissue metastasis.

## Consent

Written informed consent was obtained from the patient for publication of this Case report and any accompanying images. A copy of the written consent is available for review by the Series Editor of this journal.

## Abbreviations

MRI: Magnetic resonance images; CT: Computed tomography.

## Competing interests

The authors declare that they have no competing interests.

## Authors’ contributions

Conception and design: JI Manuscript writing: JI and SM Final approval: JI, SM, HK Pathological explorations: HK Patient's management: JI, TS, TT, TG, KH, KA and SI. All authors read and approved the final manuscript.

## Pre-publication history

The pre-publication history for this paper can be accessed here:

http://www.biomedcentral.com/1471-2407/12/313/prepub
